# Explaining the unexplained admixture mapping signals via rare variants: the HCHS/SOL

**DOI:** 10.1093/hmg/ddag098

**Published:** 2026-09-25

**Authors:** Xueying Chen, Maria Argos, Bing Yu, Eric Boerwinkle, Qibin Qi, Robert Kaplan, Nora Franceschini, Tamar Sofer

**Affiliations:** Department of Biostatistics, Harvard T.H. Chan School of Public Health, 655 Huntington Ave, Boston, MA, 02115, United States; CardioVascular Institute (CVI), Beth Israel Deaconess Medical Center, Center for Life Sciences, 3 Blackfan St., Boston, MA, 02115, United States; Department of Environmental Health, School of Public Health, Boston University, 715 Albany St, Boston, MA, 02118, United States; Division of Epidemiology and Biostatistics, School of Public Health, University of Illinois Chicago, 1603 W. Taylor St, Chicago, IL, 60612, United States; Department of Epidemiology, School of Public Health, The University of Texas Health Science Center at Houston, 1200 Pressler St, Houston, TX, 77030, United States; Department of Epidemiology, School of Public Health, The University of Texas Health Science Center at Houston, 1200 Pressler St, Houston, TX, 77030, United States; Department of Epidemiology & Population Health, Albert Einstein College of Medicine, 1300 Morris Park Avenue, Bronx, NY, 10461, United States; Department of Epidemiology & Population Health, Albert Einstein College of Medicine, 1300 Morris Park Avenue, Bronx, NY, 10461, United States; Division of Public Health Sciences, Fred Hutchinson Cancer Center, 1100 Fairview Ave. N., Seattle, WA, 98109, United States; Department of Genetics, University of North Carolina at Chapel Hill, 120 Mason Farm Road, Chapel Hill, NC, 27599, United States; Department of Epidemiology, University of North Carolina at Chapel Hill, 135 Dauer Drive, Chapel Hill, Chapel Hill, NC, 27599, United States; Department of Biostatistics, Harvard T.H. Chan School of Public Health, 655 Huntington Ave, Boston, MA, 02115, United States; CardioVascular Institute (CVI), Beth Israel Deaconess Medical Center, Center for Life Sciences, 3 Blackfan St., Boston, MA, 02115, United States; Department of Medicine, Harvard Medical School, 25 Shattuck Street, Boston, MA, 02115, United States

**Keywords:** genetic ancestry, rare variant association, common variants

## Abstract

In admixed populations, formed by a mixing of two or more previously isolated populations, genomic segments can be traced to their ancestral populations (‘ancestries’). Admixture mapping (AM) associates local ancestry with outcomes in admixed populations, detecting signals when causal variants differ in frequency or effect across ancestral populations. Prior work showed that adjusting for nearby GWAS-identified common variants does not fully explain some AM signals. Here, we assessed two approaches to explain the previously unexplained AM signal: (1) including sets of rare variants; (2) increasing the genomic region considered when searching for common variants. We studied these hypotheses comprehensively using a whole-genome sequencing dataset coupled with metabolomics from the Hispanic Community Health Study/Study of Latinos. From 16 genomic regions of interest, we identified 35 rare-variant sets associated with metabolite levels, of which 4 associations replicated. The locations of identified rare variant sets are either within the same gene or in proximity to adjusted common variants. Overall, our study provides new insight into the genetic architecture underlying AM signals via evaluating the contributions of common and rare variant sets. By integrating rare-variant analysis within the AM framework, our work highlights the potential interplay between variant types and establishes a novel strategy to guide future fine-mapping and causal variant identification in admixed populations. Yet the detected rare variants appear to explain only a small fraction of the AM signal, while inclusion of common variants from a larger genomic region appears to explain most of the AM signals.

## Introduction

Admixture refers to the interbreeding of previously isolated ancestral populations, resulting in a new population in which genomic segments can be attributed to specific ancestral origins [1]. Admixed individuals, for instance, those from Hispanic/Latino populations, possess genetic material from a combination of multiple ancestries (primarily European, African, and Amerindian ancestral populations), contributing to unique linkage disequilibrium (LD) patterns both between and within Hispanic/Latino populations [1, 2]. Additionally, allele frequencies associated with disease risk or quantitative traits often differ across these ancestral groups. The underrepresentation of Hispanic/Latino individuals in genetic association studies [3, 4], along with the complexities introduced by admixture, poses challenges for identifying causal variants and accurately estimating their effect sizes.

To address these challenges, some studies focusing on admixed populations leverage local ancestry information to identify potential variant associations with phenotype traits via admixture mapping (AM) [5–9]. Local ancestry is defined at the variant or genomic segment level and refers to the reference ancestry from which the locus was inherited. At a given position, the number of chromosomal copies inherited from a given ancestry (0, 1, or 2) is termed the local ancestry count and is used in association analyses with the phenotype as the exposure measure [10]. Local ancestry can be inferred through reference panels consisting of different populations [11, 12]. In particular, Fast Local Ancestry Estimation (FLARE) is a recently developed method using an extended Li and Stephens model with high computational efficiency [13], and has been successfully applied to multiple admixed populations [14], including participants from the Hispanic Community Health Study/Study of Latinos (HCHS/SOL).

In AM, an association is observed when either the causal variant frequencies or effect sizes (or both) differ between the modeled ancestral populations [15, 16]. AM offers greater power than standard genome-wide association studies (GWAS) by potentially capturing more complex associations, such as rare variants (RVs) and haplotypes, while also reducing the burden of multiple testing [16, 17]. However, the modeled local ancestry unit typically tags the causal variant through LD, which often extends over a broad genomic region [18], so a statistically significant signal from AM can cover many genetic variants, making pinpointing the divergence-driving variants challenging. A standard approach for identifying the causal variant is to align the AM results with the GWAS results [19]. After finding the highly significant variants in GWAS, they can be adjusted in a regression checking the association with local ancestry counts, such that we can know which variants are driving the ancestral difference. However, this approach does not always successfully fully account for the observed association. A prior study using HCHS/SOL data conducted AM between local ancestry and metabolites, identifying ancestry-enriched genomic regions associated with metabolite levels. These regions were then mapped to relevant genes and common variants, and the local ancestry-metabolite associations were adjusted to the suggested common variants. The AM signal sometimes would become non-significant in this conditional analysis but other times we were unable to identify specific genetic variants responsible for the observed associations—that is, common variants explaining the significant AM signals [19].

In GWAS, while common variants account for many phenotype associations [20], studying RVs remains essential. Recent advances in sequencing technology have made it possible to investigate RVs with minor allele frequencies (MAF) < 0.01, which were previously difficult to detect and imputed with lower accuracy [21, 22]. RVs have been reported to have important roles in complex traits and can exhibit large effect sizes [23–25], as some of the variants (e.g. putative loss-of-function and missense mutations) tend to be deleterious and occur at low allele frequencies [26, 27]. However, the potential role of RVs in relation to AM and local ancestries has not yet been thoroughly explored. Therefore, in this study we investigated RVs and their potential role in explaining the AM signal. We hypothesize that some of the unexplained AM-based associations with metabolites are due to RVs that are potentially ancestry-enriched, i.e. are more likely to be on a genomic interval inherited from one of the parental ancestries in the admixed population. Identifying ancestry-enriched RVs and their associations with metabolites can improve our understanding of the genetic basis of metabolic pathways and related diseases, especially in populations underrepresented in genetic studies. This approach may also aid in uncovering ancestry-specific risk factors and potential targets for drug development.

## Results

### Participant characteristics

Participant characteristics for those included in the metabolomics analysis are summarized in Supplementary Table S1, stratified by metabolomics batch. The average age was approximately 46 years in the discovery batch (batch 1) and 53 years in the replication batch (batch 2). Compared to batch 1, batch 2 included a higher proportion of female participants (~63% vs. ~ 57%), a greater prevalence of hypertension and/or diabetes, and lower average eGFR levels (Supplementary Table S1).

Principal components (PCs) were computed using combined data from both metabolomics batches, and the first five PCs captured most of the genetic variation and accounted for population substructure (Supplementary Fig. S1). Notably, starting from PC5 and extending through PC32, a clustering pattern emerged among self-identified Mexican individuals (Supplementary Fig. S1), which may reflect the influence of small sample sizes and the fine-scale population structure captured by whole genome sequencing (WGS) data. Overall, we used first five PCs, consistent with previous findings and established methodologies [28].

### Single variant and rare variant sets identified by the modified STAAR pipeline

Following the workflow (Fig. 1), for each metabolite and association region of interest we defined a set of ‘associated common variants’ by (1) identifying a set of GWAS-significant variants in our own analysis (2) identifying previously-reported GWAS significant variants, and (3) combining the previously-reported variants with GWAS-significant variants from our own analysis passing conditional analysis adjusting for the previously reported variants.

**Figure 1:**
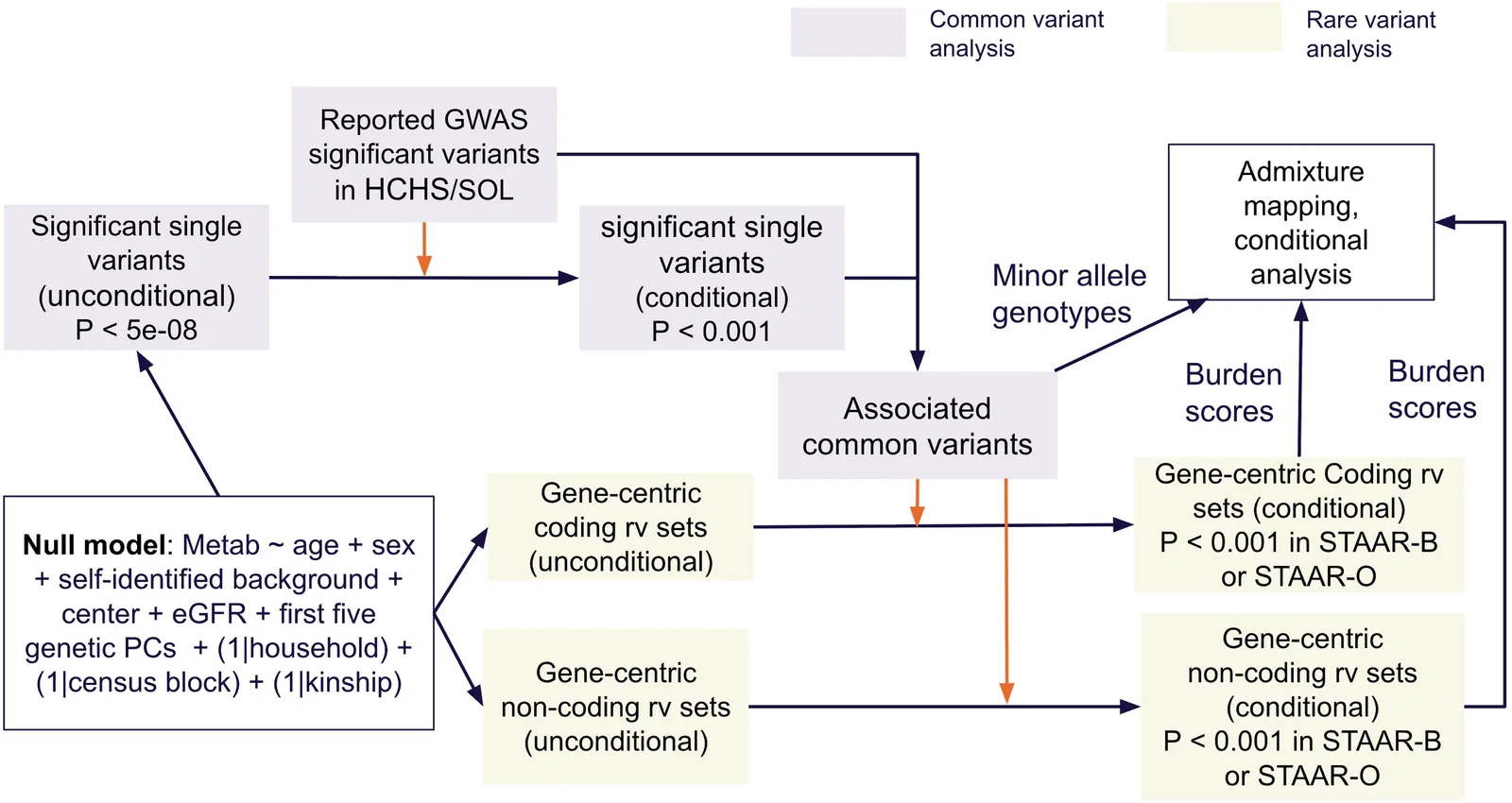
Workflow for identifying common and/or rare variant sets explaining an AM signal using the adapted STAAR pipeline. First starting with fitting the null model: Metabolite value as outcome, adjusting covariates shown above and random effects of household, census block, and kinship. Next steps are color-coded by variant type: Lavender for common variant analysis and yellow for RV analysis. Orange arrows indicate conditional analyses that adjust for the variants or sets in the target box. The significance thresholds for gene-centric unconditional analyses (both coding and non-coding) were computed by *P* < 0.05/(the number of genes × the number of function categories). For all conditional variant associations, a nominal *P*-value (0.001) was used. Conditional admixture mapping was performed using four models per metabolite–region pair: Null, common variants only, RV burden only, and a combined model including both. PC: Principal component; eGFR: Estimated glomerular filtration rate; AM: Admixture mapping; RV: Rare variant; STAAR-B: STAAR burden *P*-value; STAAR-O: STAAR omnibus *P*-value.

In the gene-centric unconditional analysis (both coding and non-coding), we identified significant RV sets, for any of the STAAR-O, STAAR-B (1,1), or STAAR-B (1, 25) *P*-value, in two negative control regions and eight test regions (Tabe 1, Supplementary Data). After adjusting the associated common variants, RV sets in 9 out of 16 regions persisted (Table 1). Among rare variant sets sharing the same functional category within the same genomic region, 4 of the 9 regions showed replication of one or more rare variant sets (Supplementary Table S2).

**Table 1:** Number of significant single common variants and rare variant sets identified using the modified STAAR pipeline, both conditional and unconditional on the ‘associated common variants’ for each trait and genomic region, which were generated by combining the variants from the 6^th^ and 7^th^ column in the table together. Afr: African ancestry, Amer: Amerindian ancestry, RV: Rare variant.

Metabolite name	Type	Chr	Region (bp)	Driving ancestry	# previously reported independent variants	# significant individual variants, (conditional *P* < 0.001), pruned	# significant RV sets, coding (unconditional, *P* < 2.52 × 10^−5^)	# unique significant RV sets, coding (conditional, *P* < .001)	# significant RV sets, non-coding (unconditional, *P* < 1.63 × 10^−5^)	# significant RV sets, non-coding (conditional, *P* < .001)
N2-acetyllysine	Negative Control	2	71 934 335–74 868 682	Afr	1	6	3	8	4	0
Betaine	Negative Control	5	78 778 375–79 374 979	Afr	2	0	0	—	0	—
N-acetylglucosaminylasparagine	Negative Control	6	167 190 769–167 300 620	Amer	2	0	1	0	0	—
Cysteinylglycine	Negative Control	16	87 530 462–90 083 514	Amer	1	2	0	—	0	—
N-acetylarginine	Test Region	2	71 934 335–74 810 716	Afr	3	7	6	0	16	1
3-aminoisobutyrate	Test Region	5	34 648 620–35 360 348	Amer	5	5	5	2	7	0
N-acetylputrescine	Test Region	8	17 749 979–18 453 195	Amer	3	3	0	—	0	—
5-oxoproline	Test Region	8	141 782 700–144 007 080	Afr	2	6	6	2	12	0
Carnitine	Test Region	10	59 301 068–61 869 652	Amer	2	3	0	—	0	—
Arachidonoylcholine	Test Region	11	60 178 829–65 895 768	Amer	1	8	1	1	0	—
3beta-hydroxy-5-cholestenoate	Test Region	11	107 710 259–111 394 190	Amer	2	3	0	—	0	—
1-methylimidazoleacetate	Test Region	12	84 652–502 428	Amer	2	4	3	2	0	—
Ethylmalonate	Test Region	12	120 613 482–122 018 618	Afr	2	0	3	2	0	—
N-acetylcarnosine	Test Region	13	95 131 902–96 203 528	Afr	3	0	0	—	0	—
Propyl 4-hydroxybenzoate sulfate	Test Region	16	27 060 560–31 617 061	Afr	3	15	5	0	31	14
Cys-gly, oxidized	Test Region	16	87 530 462–90 083 514	Amer	5	6	1	3	1	0

Rare variant sets identified in coding regions were observed in 7 of the 9 regions (Tables 1 and 2). Notably, *AGXT2* (chromosome 5, 3-aminoisobutyrate), *OPLAH* (chromosome 8, 5-oxoproline), and *ACADS* (chromosome 12, ethylmalonate) exhibited concordant RV sets with identical functional categories, predominantly disruptive missense variants and putative loss-of-function variants (Table 2, Supplementary Table S3).

**Table 2:** Significant rare variant sets and their associated functional categories identified using the modified STAAR pipeline, conditioned on previously associated common variants, for each metabolite and genomic region. The reported *P*-values are omnibus *P*-values derived from multiple rare variant tests (SKAT, burden, and ACAT), with the final integrated *P*-value summarized as STAAR-O. STAAR-S: The omnibus *P*-value for all SKAT tests under the specified Beta distribution. STAAR-B: The omnibus *P*-value for all burden tests under the specified Beta distribution. STAAR-A: The omnibus *P*-value for all ACAT-V tests under the specified Beta distribution. ACAT-O: The omnibus *P*-value for all aggregated tests including the SKAT, burden, and ACAT-V tests across two Beta distributions using Cauchy method. STAAR-O: The omnibus *P*-value for all aggregated tests including the SKAT, burden, and ACAT-V tests weighted by annotation using Cauchy method.

Gene name	Chr	Metabolite	Group	Category	#SNV	cMAC	STAAR-S (1,25)	STAAR-S (1,1)	STAAR-B (1,25)	STAAR-B (1,1)	STAAR-A (1,25)	STAAR-A (1,1)	ACAT-O	STAAR-O
AUP1	2	N2-acetyllysine	Negative Control	plof_ds	10	63	1.48E-03	1.46E-03	7.84E-04	6.74E-04	1.38E-03	1.37E-03	1.02E-03	1.08E-03
CYP26B1	2	N2-acetyllysine	Negative Control	missense	31	135	2.06E-02	3.61E-02	1.41E-02	1.59E-02	1.80E-04	2.03E-04	5.33E-04	5.62E-04
ALMS1	2	N2-acetyllysine	Negative Control	missense	238	974	1.38E-08	1.17E-07	2.35E-01	1.38E-01	1.47E-04	1.51E-04	2.17E-07	7.41E-08
NAT8	2	N2-acetyllysine	Negative Control	missense_cond	22	56.06	3.90E-05	3.83E-05	1.69E-03	1.27E-03	3.51E-05	3.48E-05	5.43E-05	5.43E-05
AUP1	2	N2-acetyllysine	Negative Control	disruptive_missense_cond	9	62	1.47E-03	1.45E-03	9.12E-04	7.81E-04	1.39E-03	1.37E-03	1.10E-03	1.15E-03
EGR4	2	N2-acetyllysine	Negative Control	synonymous_cond	18	81	4.39E-06	4.30E-06	1.10E-03	5.76E-04	3.97E-06	3.92E-06	6.21E-06	6.19E-06
ALMS1	2	N2-acetyllysine	Negative Control	synonymous_cond	81	603	1.36E-06	2.45E-06	9.00E-01	8.10E-01	7.94E-04	6.82E-04	6.81E-06	5.24E-06
STAMBP	2	N2-acetyllysine	Negative Control	synonymous	7	82	1.13E-03	1.28E-03	1.69E-04	1.60E-04	1.86E-03	1.82E-03	3.80E-04	4.03E-04
DUSP11	2	N-acetylarginine	Test Regions	enhancer_CAGE	10	16	4.42E-05	4.31E-05	3.00E-01	2.90E-01	3.00E-01	2.90E-01	1.33E-04	1.31E-04
AGXT2	5	3-aminoisobutyrate	Test Regions	plof_ds	15	58	5.30E-06	6.51E-06	8.40E-11	1.10E-10	8.45E-06	8.98E-06	1.57E-10	2.86E-10
AGXT2	5	3-aminoisobutyrate	Test Regions	disruptive missense	11	52	4.54E-06	5.74E-06	7.37E-11	9.82E-11	5.14E-06	5.73E-06	1.85E-10	2.53E-10
OPLAH	8	5-oxoproline	Test Regions	plof_ds	14	23	1.22E-02	1.25E-02	3.83E-05	3.91E-05	3.83E-05	3.91E-05	5.09E-05	5.79E-05
OPLAH	8	5-oxoproline	Test Regions	disruptive missense	11	19	9.19E-03	9.43E-03	1.12E-04	1.14E-04	1.12E-04	1.14E-04	1.64E-04	1.69E-04
MS4A14	11	Arachidonoylcholine	Test Regions	synonymous	25	175	4.96E-03	6.28E-03	1.24E-04	1.42E-04	1.51E-02	1.43E-02	3.57E-04	3.85E-04
SLC6A13	12	1-methylimidazoleacetate	Test Regions	plof_ds	20	71	1.12E-04	1.28E-04	1.07E-05	8.90E-06	7.01E-04	6.77E-04	2.59E-05	2.66E-05
SLC6A13	12	1-methylimidazoleacetate	Test Regions	disruptive_missense	18	69	1.13E-04	1.31E-04	5.17E-06	4.41E-06	7.00E-04	6.77E-04	1.28E-05	1.37E-05
ACADS	12	Ethylmalonate	Test Regions	plof_ds	26	34	7.56E-08	7.75E-08	6.26E-10	6.09E-10	6.26E-10	6.09E-10	3.37E-09	9.23E-10
ACADS	12	Ethylmalonate	Test Regions	disruptive_missense	25	33	6.26E-08	6.42E-08	3.02E-10	2.95E-10	3.02E-10	2.95E-10	9.09E-10	4.47E-10
FAM57B	16	Propyl 4-hydroxybenzoate sulfate	Test Regions	enhancer_DHS	66	288	2.60E-03	3.12E-03	5.96E-05	4.45E-05	3.33E-02	3.28E-02	1.17E-04	1.50E-04
FUS	16	Propyl 4-hydroxybenzoate sulfate	Test Regions	enhancer_DHS	123	778	3.51E-02	3.77E-02	2.79E-04	1.78E-04	1.22E-01	1.19E-01	6.31E-04	6.48E-04
IL4R	16	Propyl 4-hydroxybenzoate sulfate	Test Regions	UTR	55	425	4.24E-05	2.20E-05	2.50E-03	1.12E-03	6.00E-05	4.98E-05	5.70E-05	5.61E-05
IL4R	16	Propyl 4-hydroxybenzoate sulfate	Test Regions	promoter_CAGE	33	162	3.01E-06	2.94E-06	7.74E-04	3.04E-04	5.25E-06	4.77E-06	5.26E-06	5.57E-06
IL4R	16	Propyl 4-hydroxybenzoate sulfate	Test Regions	promoter_DHS	84	663	3.81E-07	4.30E-07	2.64E-04	1.42E-04	8.84E-05	7.68E-05	7.35E-07	1.20E-06
IL4R	16	Propyl 4-hydroxybenzoate sulfate	Test Regions	enhancer_CAGE	92	479	4.53E-05	3.53E-05	1.05E-02	8.30E-03	5.85E-05	5.01E-05	6.05E-05	6.84E-05
IL4R	16	Propyl 4-hydroxybenzoate sulfate	Test Regions	enhancer_DHS	507	3001	1.73E-03	6.48E-04	6.17E-04	4.83E-04	3.01E-04	2.52E-04	5.12E-04	4.58E-04
ITGAL	16	Propyl 4-hydroxybenzoate sulfate	Test Regions	promoter_DHS	54	145	2.78E-03	2.95E-03	6.65E-04	5.82E-04	2.39E-02	2.49E-02	1.63E-03	1.50E-03
ITGAL	16	Propyl 4-hydroxybenzoate sulfate	Test Regions	enhancer_DHS	63	261	5.72E-02	6.59E-02	1.27E-04	1.34E-04	7.60E-02	8.20E-02	4.01E-04	3.90E-04
KDM8	16	Propyl 4-hydroxybenzoate sulfate	Test Regions	enhancer_DHS	194	1078	1.76E-04	2.48E-04	4.52E-02	2.77E-02	2.14E-02	2.03E-02	9.76E-04	6.09E-04
MVP	16	Propyl 4-hydroxybenzoate sulfate	Test Regions	UTR	26	115	4.11E-04	4.42E-04	8.42E-02	4.35E-02	5.21E-04	5.15E-04	7.03E-04	6.99E-04
NUPR1	16	Propyl 4-hydroxybenzoate sulfate	Test Regions	promoter_DHS	6	43	5.41E-04	5.48E-04	9.14E-03	7.47E-03	6.88E-04	6.77E-04	8.71E-04	8.76E-04
PRRT2	16	Propyl 4-hydroxybenzoate sulfate	Test Regions	enhancer_DHS	294	1078	1.46E-05	3.88E-05	9.36E-03	6.37E-03	4.30E-03	4.32E-03	6.13E-05	6.33E-05
RNF40	16	Propyl 4-hydroxybenzoate sulfate	Test Regions	UTR	31	119	7.45E-05	9.75E-05	3.56E-03	3.23E-03	2.15E-03	2.15E-03	1.63E-04	2.38E-04
DPEP1	16	Cys-gly, oxidized	Test Regions	plof	3	4	5.76E-03	5.80E-03	3.73E-04	3.74E-04	3.73E-04	3.74E-04	4.28E-04	5.43E-04
DPEP1	16	Cys-gly, oxidized	Test Regions	plof_ds	6	10	6.90E-04	7.19E-04	5.38E-07	5.47E-07	5.38E-07	5.47E-07	5.88E-07	8.13E-07
DPEP1	16	Cys-gly, oxidized	Test Regions	disruptive_missense	3	6	5.24E-03	5.33E-03	3.77E-04	3.80E-04	3.77E-04	3.80E-04	5.15E-04	5.48E-04

From the non-coding analysis, RV sets were identified at two test regions (Table 1 and 2). The region at chromosome 16 associated with propyl 4-hydroxybenzoate sulfate included three RV sets that replicated in batch 2 after conditioning on the associated common variants: *FAM57B* and *PRRT2* (enhancer region that overlaps DHS site), and *RNF40* (UTR region) (Supplementary Table S3). The limited number of RV sets identified after conditioning on common variants suggests that, across all 16 selected genomic regions, most of the genetic associations were largely accounted for by the common variants.

### Admixture mapping with rare variants—Negative controls

After adjusted for the ‘associated common variants’, the AM signals were substantially reduced across the four negative control regions, and became statistically insignificant (Fig. 2, Table 3). In batch 2, three out of four AM signals displayed patterns consistent with batch 1. The African local ancestry association with betaine did not replicate, even without conditioning on any common variant genotypes (Fig. 2, Supplementary Table S4). We identified eight significant RV sets in one of the negative controls on chromosome 2 in batch 1, spanning multiple genes and functional categories: missense or disruptive missense variants (*AUP1, ALMS1, NAT8, CYP26B1*), plof_ds (*AUP1*), and synonymous variants (*EGR4, ALMS1, STAMBP*) (Table 2). However, conditional admixture mapping incorporating the corresponding burden scores resulted in only a partially reduced the admixture signal, which remained above the significance threshold (Fig. 2, Table 3). None of the RV sets mentioned above were replicated in batch 2 (Supplementary Tables S2 and S3).

**Figure 2:**
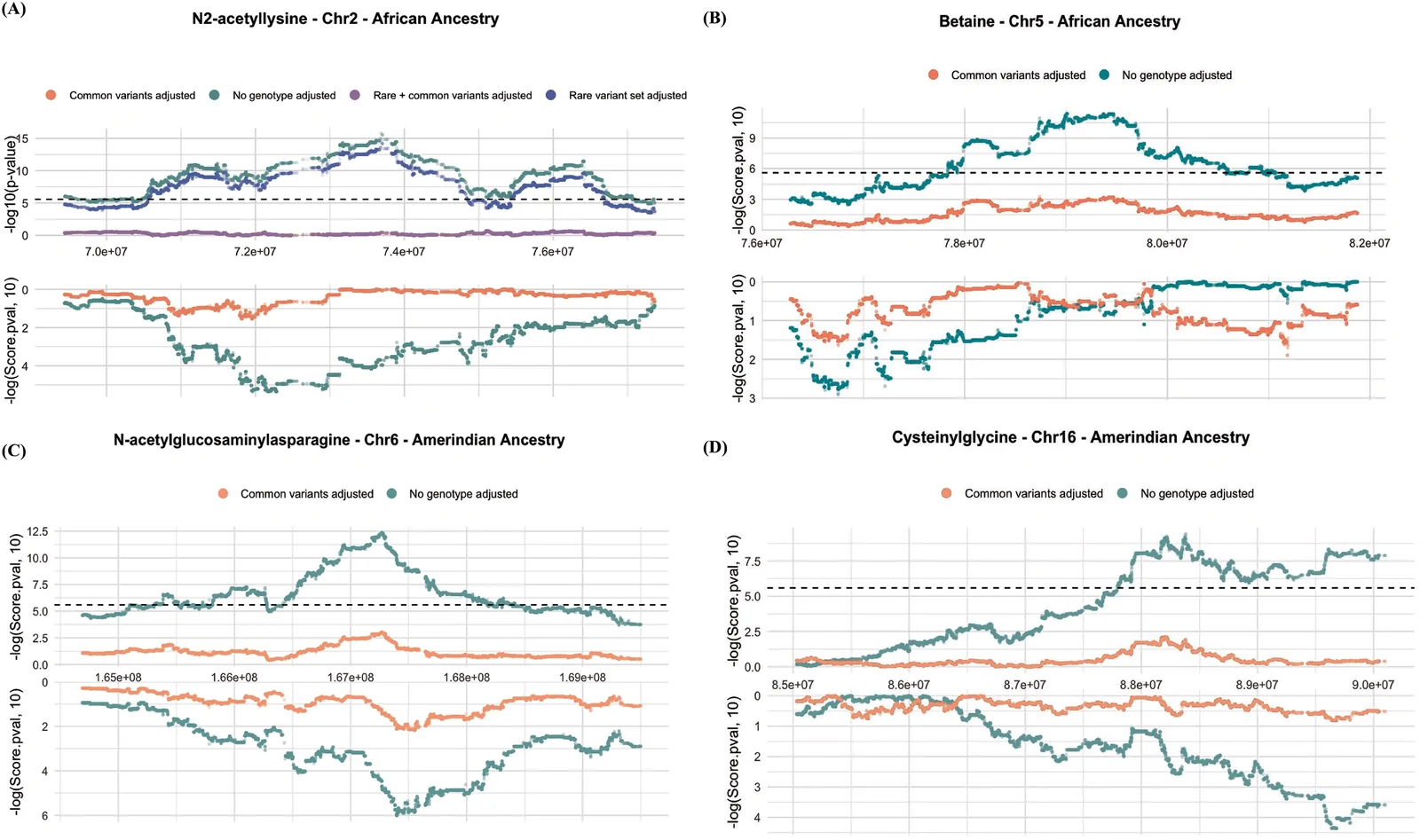
A.M. results in terms of -log10(P) using the driving local ancestries for four ‘negative control’ associations, defined as those with associations explained by common variants or rare variant sets (if identified): (A) N2-acetyllysine on chromosome 2, (B) betaine on chromosome 5 (both driven by African ancestry), (C) N-acetylglucosaminylasparagine on chromosome 6, and (D) cysteinylglycine on chromosome 16 (both driven by Amerindian ancestry). For each trait, mirrored plots display results from the discovery dataset (batch 1, upper panel) and the replication dataset (batch 2, lower panel). The horizontal dashed black line in the upper panel denotes the AM significance threshold (*P* = 2.58 × 10^−6^).

**Table 3:** A.M. results for all 16 metabolite–genomic region pairs are shown, including effect size estimates and *P*-values of the lead AM variant under four models: The null model (no genotype adjustment), adjustment for associated common variants only, adjustment for rare variant sets using burden scores, and adjustment for both common and rare variants (if a rare variant set was identified). RV: Rare variant.

Metabolite name	Group	Chr	Region (bp)	Driving ancestry	AM lead variant Est. null model	AM lead variant *P*-value null model	AM lead variant Est. adjusting associated common variants	AM lead variant *P*-value adjusting associated common variants	AM lead variant Est. adjusting RV sets only	AM lead variant *P*-value adjusting RV sets only	AM lead variant Est. adjusting RV sets + associated common variants	AM lead variant *P*-value adjusting RV sets + associated common variants
N2-acetyllysine	Negative Control	2	71 934 335–74 868 682	Afr	0.15	1.60E-16	0.01	3.30E-03	0.04	3.53E-15	0.01	3.28E-03
Betaine	Negative Control	5	78 778 375–79 374 979	Afr	−0.07	4.19E-12	−0.02	5.60E-04	–	–	–	–
N-acetylglucosaminylasparagine	Negative Control	6	167 190 769–167 300 620	Amer	−0.11	4.61E-13	−0.02	1.00E-03	–	–	–	–
Cysteinylglycine	Negative Control	16	87 530 462–90 083 514	Amer	0.07	3.91E-10	0.01	7.43E-03	–	–	–	–
N-acetylarginine	Test Regions	2	71 934 335–74 810 716	Afr	0.04	6.6E-22	−0.01	2.02E-03	0.05	5.62E-22	−0.01	2.24E-03
3-aminoisobutyrate	Test Regions	5	34 648 620–35 360 348	Amer	0.05	3.04E-31	−0.01	6.37E-03	0.05	1.58E-32	−0.01	6.37E-03
N-acetylputrescine	Test Regions	8	17 749 979–18 453 195	Amer	0.02	5.99E-08	−0.01	1.10E-02	–	–	–	–
5-oxoproline	Test Regions	8	141 782 700–144 007 080	Afr	−0.10	4.38E-25	0.01	5.90E-03	−0.05	2.18E-25	0.01	3.78E-03
Carnitine	Test Regions	10	59 301 068–61 869 652	Amer	0.07	6.25E-17	−0.01	5.49E-03	–	–	–	–
Arachidonoylcholine	Test Regions	11	60 178 829–65 895 768	Amer	−0.04	6.2E-30	−0.02	7.66E-03	−0.04	2.87E-29	−0.02	7.66E-03
3beta-hydroxy-5-cholestenoate	Test Regions	11	107 710 259–111 394 190	Amer	−0.04	5.11E-27	−0.02	5.01E-05	–	–	–	–
1-methylimidazoleacetate	Test Regions	12	84 652–502 428	Amer	−0.04	1.38E-29	−0.01	9.91E-03	−0.04	1.74E-28	−0.01	4.86E-03
Ethylmalonate	Test Regions	12	120 613 482–122 018 618	Afr	−0.04	3.24E-15	0.01	5.04E-03	−0.04	2.96E-16	0.01	7.12E-03
N-acetylcarnosine	Test Regions	13	95 131 902–96 203 528	Afr	0.05	3.26E-09	0.01	6.51E-03	–	–	–	–
Propyl 4-hydroxybenzoate sulfate	Test Regions	16	27 060 560–31 617 061	Afr	0.06	4.63E-34	0.03	7.75E-06	0.05	4.65E-19	0.03	7.75E-06
Cys-gly, oxidized	Test Regions	16	87 530 462–90 083 514	Amer	0.05	9.42E-34	−0.01	4.27E-03	0.05	4.78E-34	−0.01	4.99E-03

We also examined the span of associated common variants adjusted across the significant AM region. Most of the common variants are in high proximity to each other, while two variants at chromosome 2 are situated over 1.5 Mb away but are still significantly associated with the metabolites in conditional analysis (Fig. 4A).

### Admixture mapping with rare variants—Test regions

For the genomic regions whose AM signals were not explained in the previous study (referred to as test regions), we first adjusted for the ‘associated common variants’ identified through single variant analysis in combination of known genetic associations to assess how much of the admixture mapping (AM) signal could be explained and whether the results aligned with previously reported findings. In all twelve test regions, the AM signals were explained solely by adjusting for significantly associated common variants within an expanded region compared to the previous publication [19], consistently across both the discovery and replication batches (Table 3, and Supplementary Table S3).

To assess the impacts of RVs in AM, we extracted burden scores from the significant RV sets identified in the selected regions. In regions with multiple RV sets, all unique gene–category pairs that were not highly correlated (r < 0.85) were jointly included as covariates in the conditional AM through their burden scores (Supplementary Fig. S2). When only adjusting for the RV sets at the propyl 4-hydroxybenzoate sulfate on chromosome 16 test region, the AM signal in African ancestry became partially explained compared to the null model, but remained significant (Fig. 3D, Table 3). The gap observed in (D) is attributable to missing local ancestry calls in the centromeric region, likely due to exclusion during the quality-control step. However, in the remaining regions, adjusting for RV sets alone resulted in little change relative to the null model (Fig. 3A–C, Table 3, and Supplementary Fig. S3). Adjusting for both the associated common variants and the RV set burdens did not further change the AM association (Fig. 3, Table 3, and Supplementary Fig. S3). Supplementary Fig. S4 further visualizes, for each of the AM signal regions in batch 1, the estimated effect and 95% confidence interval (CI) of the lead AM variant before and after the conditional analyses. For the four regions with RV sets replicated in batch 2, the patterns were similar as those shown in batch 1 (Fig. 3).

**Figure 3:**
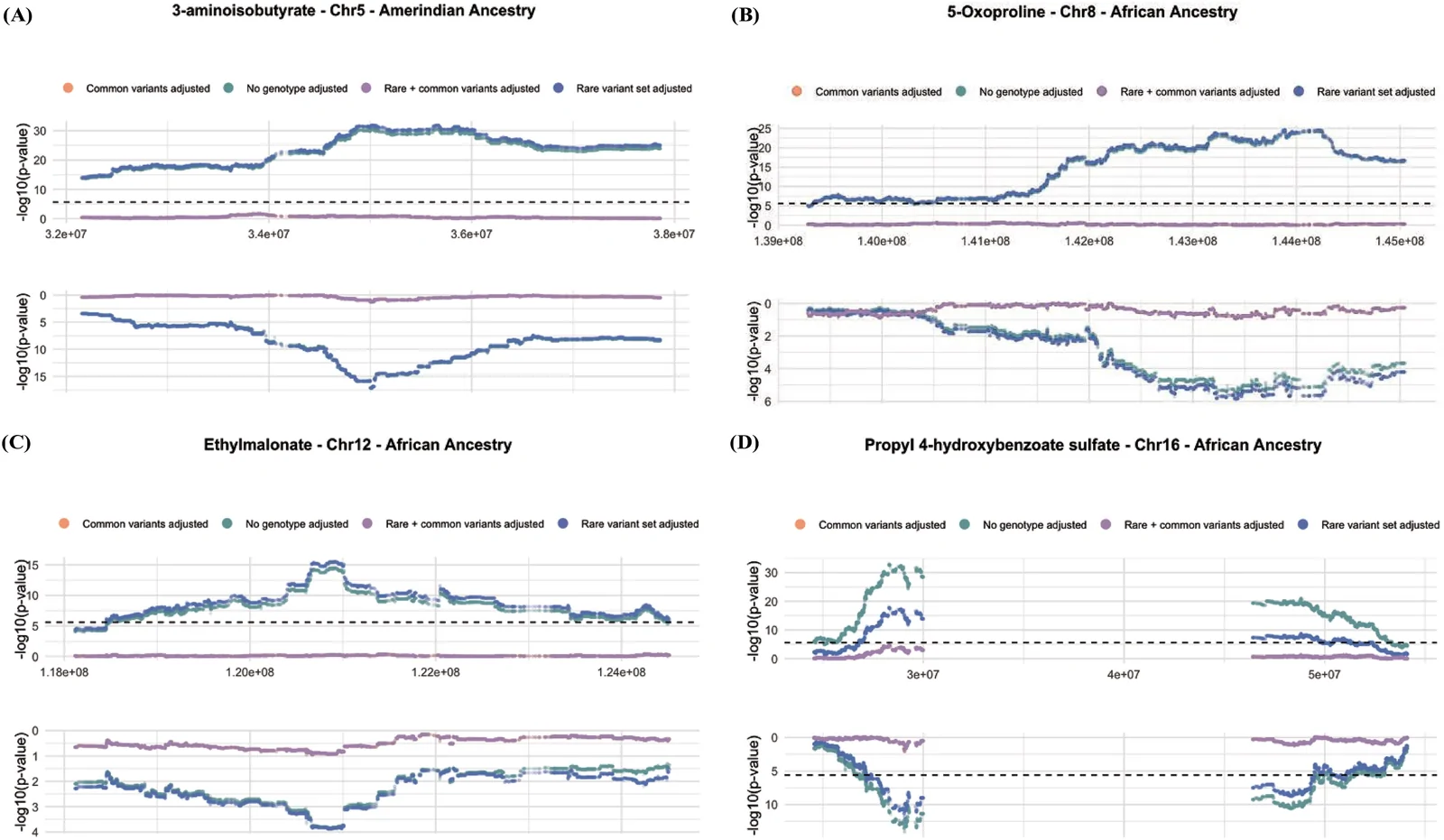
A.M. results in terms of -log10(P) using the (A) Amerindian local ancestry for 3-aminoisobutyrate on chromosome 5, (B) African local ancestry for 5-oxoproline on chromosome 8, (C) African local ancestry for ethylmalonate on chromosome 12, and (D) African ancestry for propyl 4-hydroxybenzoate sulfate on chromosome 16, using null model (no genotype adjusted), model adjusting common variants, model adjusting rare variants (RVs), and model adjusting both rare and common variants for discovery (upper) and replication batch (lower). The gap observed in (D) reflects the absence of local ancestry information in that region, which is near the centromere and was likely removed during quality control. The horizontal dashed black line in the upper panel denotes the AM significance threshold (*P* = 2.58 × 10^−6^). The RV sets included in the batch 2 were those that had also been replicated in batch 1.

**Figure 4:**
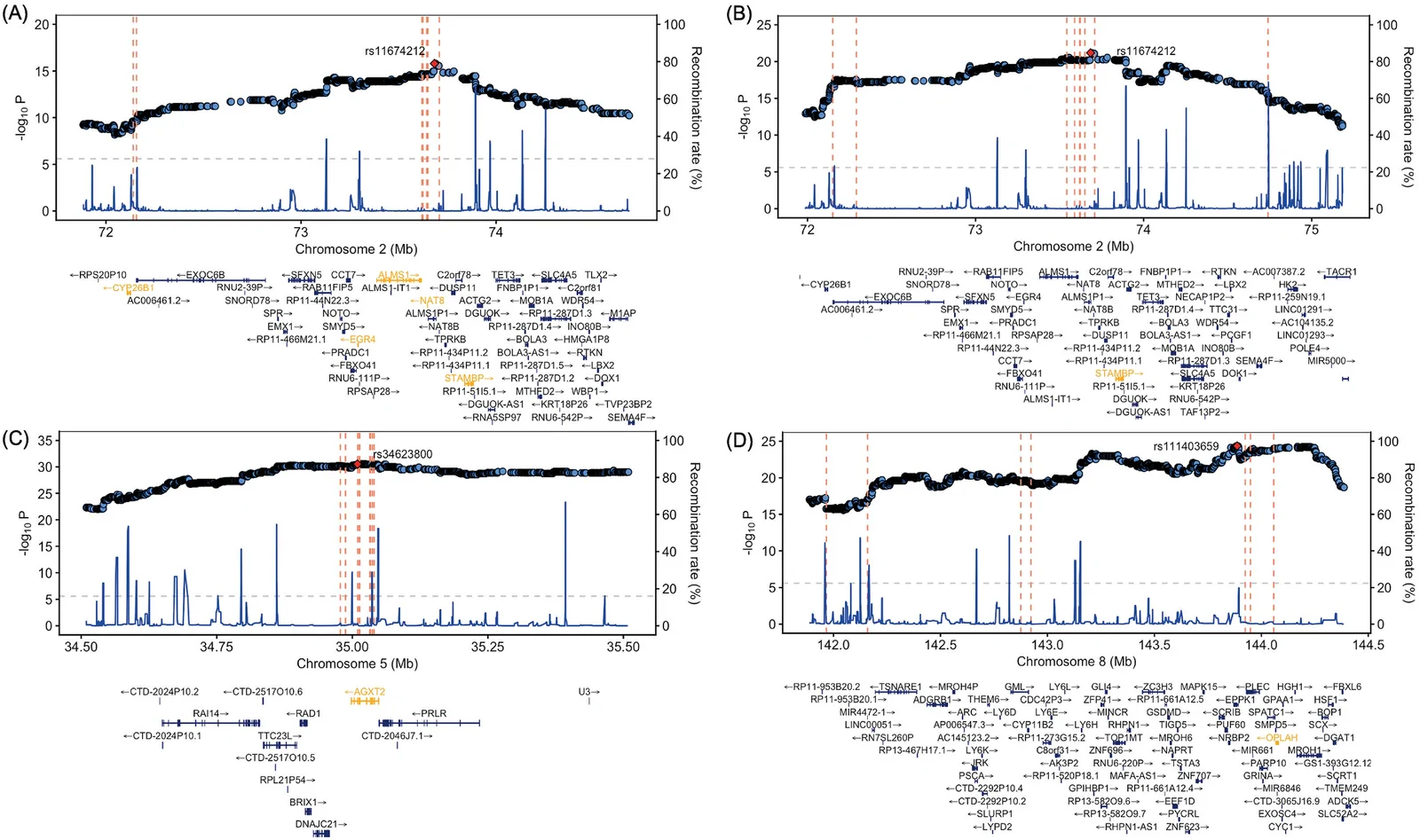
A.M. regional association plots of regions where significant rare variant sets were identified, showing positions of associated common variants and genes with significant rare variant sets in conditional analysis (chr 2–8). The horizontal dashed line shows AM significance threshold (*P* = 2.58 × 10^−6^). (A) African ancestry for N2-acetyllysine on chromosome 2, (B) African ancestry for N-acetylarginine on chromosome 2 (C) Amerindian ancestry for 3-aminoisobutyrate on chromosome 5, (D) African ancestry for 5-oxoproline on chromosome 8. Each vertical dashed line marks the position of an associated common variant, while the highlighted gene denotes the genomic location and gene assignment of the rare variant sets identified in the conditional analysis.

The genomic positions of associated common variants could span a relatively wide region (1–2 Mb) on chromosomes 2 (N-acetylarginine), 8 (5-oxoproline), 10 (carnitine), and 11 (arachidonoylcholine and 3beta-hydroxy-5-cholestenoate), while other regions spanning a narrower genomic range (Figs 4 and 5, Table 4). Among all regions with RV sets identified in conditional analysis, the mapped genes harboring these sets are very close to associated common variants (Figs 4 and 5). For example, for cys-gly (oxidized) on chromosome 16, both rare and common variants are concentrated around the *DPEP1* gene, and for 3-aminoisobutyrate on chromosome 5, around the *AGXT2* gene (Fig. 5E, Table 4).

**Figure 5:**
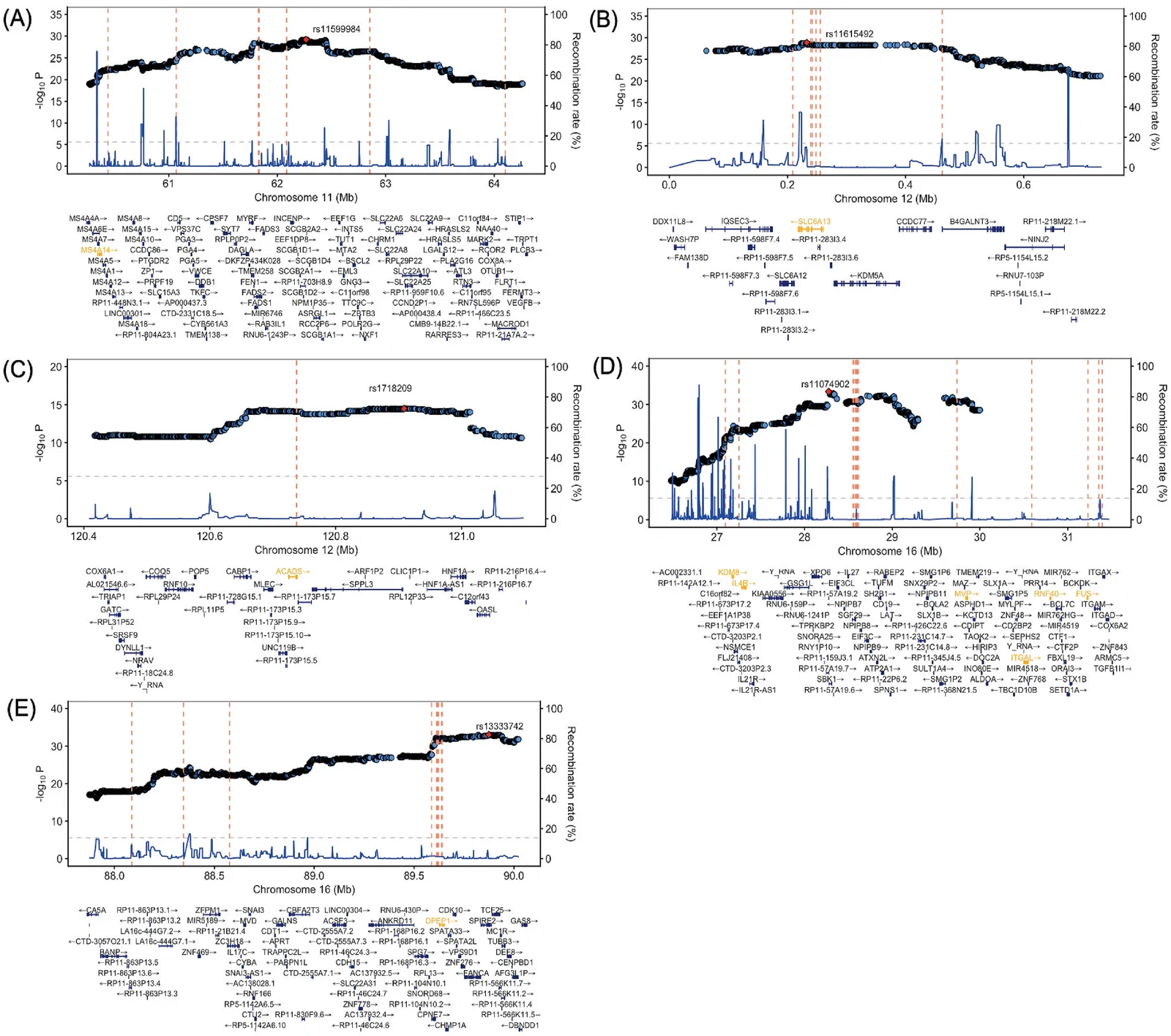
A.M. regional association plots showing positions of associated common variants and genes with significant rare variant sets in conditional analysis (chr 11–16). The horizontal dashed line shows AM significance threshold (*P* = 2.58 × 10^−6^). (A) Amerindian ancestry for arachidonoylcholine on chromosome 11, (B) Amerindian ancestry for 1-methylimidazoleacetate on chromosome 12, (C) African ancestry for ethylmalonate on chromosome 12, (D) African ancestry for propyl 4-hydroxybenzoate sulfate on chromosome 16, and (E) Amerindian ancestry for cys-gly, oxidized on chromosome 16. Each vertical dashed line marks the position of an associated common variant, while the highlighted gene denotes the genomic location and gene assignment of the rare variant sets identified in the conditional analysis.

**Table 4:** Genomic position span (in base pairs) of associated common variants across all sixteen metabolite–genomic regions.

Metabolite Name	Group	Chr	Span of associated common variants (bp)
N2-acetyllysine	Negative Control	2	1 569 130
Betaine	Negative Control	5	100 437
N-acetylglucosaminylasparagine	Negative Control	6	19 458
Cysteinylglycine	Negative Control	16	13 223
N-acetylarginine	Test Regions	2	2 589 744
3-aminoisobutyrate	Test Regions	5	62 060
N-acetylputrescine	Test Regions	8	20 302
5-oxoproline	Test Regions	8	2 091 693
Carnitine	Test Regions	10	2 412 517
Arachidonoylcholine	Test Regions	11	4 998 072
3beta-hydroxy-5-cholestenoate	Test Regions	11	2 604 299
1-methylimidazoleacetate	Test Regions	12	253 199
Ethylmalonate	Test Regions	12	405
N-acetylcarnosine	Test Regions	13	104 871
Propyl 4-hydroxybenzoate sulfate	Test Regions	16	4 504 064
Cys-gly, oxidized	Test Regions	16	1 554 066

## Discussion

Admixture mapping is effective in identifying ancestry-enriched regions associated with phenotypic traits by leveraging inferred local ancestries. Due to the extensive local ancestry regions in admixed populations and unclear mechanism of local ancestry association [29], examining how AM signals can be explained is essential for discovery of specific trait-associated genetic variants. A longstanding hypothesis has been that AM associations that were not explained by common variants are driven by rare genetic variants. This work aimed to study this hypothesis.

We identified 35 metabolite-associated RV sets with coding or non-coding functions through gene-centric analyses for 16 selected genomic regions via the modified pipeline for RV associations and replicated 4 of the RV set associations in the replication batch. We found no significantly associated RV sets in some genomic regions (Table 2), and for those with RV sets, the genes containing those RVs are close to the associated common variants (Figs 4 and 5). The number of significantly associated RV sets dropped significantly once conditioning on the associated common variants, from 104 to 35 in batch 1 and 67 to 4 in batch 2 (Table 2).

Our previous study found that regions with strong AM signals could not be explained by genome-wide significant common variants suggested by COJO analysis applied over GWAS summary statistics [19]. COJO iteratively conditions on the most strongly associated variant to detect additional independent variants, until no genome-wide significant SNPs remain [30, 31]. In the two examples from our previous study [19], the method attenuated the previously unexplained AM signals to below the STEAM significance threshold (*P* = 2.58 × 10^−6^) after covariate adjustment, though we note this reflects a loss of statistical significance rather than a complete biological explanation of the signal. This method is analogous to our workflow of fitting ‘associated common variants’ in conditional AM, but differs in the methods of identifying common variants, and the results that AM signals were explained after adjustment are consistent.

Here, we defined a genomic interval within which we jointly interrogated both common and rare variants, accounting for all AM signals across the four negative control regions and twelve test regions with previously unexplained AM signals. The corresponding attenuation in AM association strength between the unconditional and conditional AM analyses was consistently replicated in batch 2 (Table 3, Supplementary Table S3). Note that in our sensitivity analysis using a combined-pruned variants, admixture-mapping results remained consistent with our original findings (Table 3, Supplementary Table S5), with only minimal changes in effect sizes and significance and signals remaining attenuated in 14/16 regions (Supplementary Table S5). The exceptions were the region on chromosome 16 for Cys-gly, oxidized, where fewer common variants were selected (7 vs. 11), and on chromosome 12 for metabolite 1-methylimidazoleacetate (3 vs. 6). For both regions, the local ancestry signal was less attenuated in the sensitivity analysis compared to the main analysis and the AM *P*-values of the lead variants remained significant compared to the STEAM threshold (*P* < 2.58 × 10^−6^) (Supplementary Table S5).

Burden scores extracted from a single RV set had limited ability for explaining the AM signals (Table 3, Supplementary Table S3). When there were more RV sets, as in the chromosome 16 test region for propyl 4-hydroxybenzoate sulfate, we observed more signal reduction than those in other regions (Fig. 3D). Rare variant sets identified were often located near the adjusted common variants, suggesting that they are associated with the same gene (Figs 4 and 5).

Several considerations apply to the interpretation of the conditional admixture mapping analyses. Admixture mapping derives its power from the frequency differentiation of causal variants between ancestral populations, which necessarily implies correlation between local ancestry dosage and the genotypes of common variants with differential frequencies across the ancestral populations. Conditioning on the genotypes of those common variants therefore introduces collinearity by construction, and the concern is that a sufficiently large set of conditionally selected variants spanning a long ancestry tract could act as a linear proxy for the local ancestry haplotype itself, attenuating the admixture mapping signal for statistical rather than biological reasons. However, when examining the variance of local ancestry dosage explained by the associated common variants, we observed cases where six variants only explained 12% of the local ancestry variance, and cases where the single variant most significantly associated with the metabolite explained up to 22% variance of the local ancestry alone (Table 5), reflecting the substantial ancestral frequency differentiation of that variant rather than invalidity of the model. In other cases, 8 of the 16 regions, common variants explained up to 43% to 58% percent of the variance, but the range of R^2^ values across the AM signal regions were wide (Table 5).

**Table 5:** For the four negative-control and twelve test regions with significant admixture mapping (AM) signals (*P* < 2.58 × 10^−6^) in the null models, the percentage by which the local-ancestry effect on the metabolite was attenuated after adjusting for all associated common variants (median [IQR] across variants in the region), the range proportion of local-ancestry variance explained by the associated common variants (R^2^) in the region, as well as the variance explained by the top common variant that is most significantly associated with the metabolite. The range is computed over the 50 randomly selected SNP-level local ancestry counts in the metabolite-associated region. Negative values of adjusted R^2^ indicate the burden scores explain no more variance than expected by chance. Driving ancestry denotes the ancestral background underlying the AM signal (Afr, African; Amer, Amerindian).

Metabolite name	Group	Chr	Driving ancestry	Number of associated common variants	Proportion of effect size changed after adjusting associated common variants, % (median [IQR])	Proportion range of local ancestry variance explained by associated common variants (R ^2^ )	Proportion range of local ancestry variance explained by the top-associated common variant (R ^2^ )
N2-acetyllysine	Negative Control	2	Afr	7	99.01 (4.55)	(0.12, 0.56)	(−0.002, 0.05)
Betaine	Negative Control	5	Afr	2	78.38 (2.56)	(−0.02, 0.18)	(−0.03, 0.15)
N-acetylglucosaminylasparagine	Negative Control	6	Amer	2	84.84 (4.66)	(−0.03, 0.12)	(−0.05, 0.01)
Cysteinylglycine	Negative Control	16	Amer	3	89.58 (7.70)	(0.15, 0.31)	(0.04, 0.14)
N-acetylarginine	Test Regions	2	Afr	10	100.34 (14.63)	(0.06, 0.48)	(−0.01, 0.28)
3-aminoisobutyrate	Test Regions	5	Amer	10	103.32 (9.51)	(0.04, 0.27)	(−0.02, 0.01)
N-acetylputrescine	Test Regions	8	Amer	6	83.57 (7.50)	(0.02, 0.12)	(0.01, 0.09)
5-oxoproline	Test Regions	8	Afr	8	100.53 (7.10)	(0.1, 0.57)	(0.02, 0.22)
Carnitine	Test Regions	10	Amer	5	85.72 (2.65)	(0.05, 0.43)	(−0.03, 0.07)
Arachidonoylcholine	Test Regions	11	Amer	9	83.26 (11.05)	(0.12, 0.58)	(−0.02, 0.09)
3beta-hydroxy-5-cholestenoate	Test Regions	11	Amer	5	63.71 (8.94)	(0.02, 0.31)	(−0.04, 0.21)
1-methylimidazoleacetate	Test Regions	12	Amer	6	87.78 (9.90)	(0.09, 0.43)	(0.01, 0.06)
Ethylmalonate	Test Regions	12	Afr	2	100.04 (6.27)	(−0.16, 0.14)	(−0.15, 0.15)
N-acetylcarnosine	Test Regions	13	Afr	3	82.56 (3.41)	(0.06, 0.14)	(0.02, 0.12)
Propyl 4-hydroxybenzoate sulfate	Test Regions	16	Afr	18	84.39 (11.62)	(0.01, 0.50)	(−0.03, 0.07)
Cys-gly, oxidized	Test Regions	16	Amer	11	82.15 (13.77)	(0.08, 0.45)	(0.02, 0.12)

We also assessed the percentage change in local ancestry effect size estimates between the null model and the model adjusting associated common variants. In most regions, the median percentage changes were approximately 85% across all regions (range 64%–103%) (Table 5), indicating that effect size estimates shifted toward the null—in some cases crossing it (values exceeding 100%, reflecting a sign flip). If this loss of significance were driven by inflated standard errors, we would not expect to observe percentage changes of this magnitude in the effect sizes themselves. Supplementary Fig. S4 provides additional visualization addressing both the change in estimated AM effect and potential variance inflation: it provides the effect size estimates and 95% CIs for the top AM variant from each region, showing that 95% CIs from common variant-conditional AM models were of similar width, while substantially shifted towards the null, compared to those from the unadjusted models (Supplementary Fig. S4). We therefore conclude that the loss of statistical significance is a valid observation rather than an artifact of inflation.

Correlation between local ancestry and the conditioned variants is necessary but not sufficient to attenuate an admixture mapping signal. The shift in the local ancestry effect upon conditioning is the product of the variant’s association with local ancestry and its association with the metabolite. Nevertheless, we recognize the limitation of collinearity in conditional AM analysis that adjusts directly for genotypes. The common variant genotypes are by design partially collinear with the exposure of interest, and no purely statistical criterion cleanly separates a signal that is accounted for by the conditioned variants from one that has been absorbed by their correlation with ancestry. The limitation should be fully acknowledged during interpretation for such conditional AM analyses.

We similarly examined potential collinearity between the RV burden scores and the local ancestry counts in the AM signal regions, to assess the potential for variance inflation. In each AM signal region, we regressed SNP-level local ancestry counts (in statistically significant loci) over the burden scores included jointly as covariates and over the burden score most strongly associated with the metabolite alone (Table 6). In the AM model (metabolite levels regressed over local ancestry counts and adjusting covariates), the variance of the local ancestry coefficient is affected by the multiple correlation between the local ancestry and all remaining predictors, not by collinearity among those predictors themselves [32]. Because the R^2^ values were relatively low, mutual correlation among the burden scores is not expected to inflate the standard error of the local ancestry dosage term.

**Table 6:** For the four negative-control and twelve test regions with significant admixture mapping (AM) signals (*P* < 2.58 × 10^−6^) in the null models, the range proportion of local-ancestry variance explained by the burden scores from all significantly associated rare variant (RV) sets (R^2^) within the region, as well as the variance explained by the burden scores from the rare variant set that is most significantly associated with the metabolite. The range is computed over the 50 randomly selected SNP-level local ancestry counts in the metabolite-associated region. Negative values of adjusted R^2^ indicate the burden scores explain no more variance than expected by chance. ‘—’ Means the region contained a single RV set, so the two models coincide. Driving ancestry denotes the ancestral background underlying the AM signal (Afr, African; Amer, Amerindian).

Metabolite name		Group	Chr	Driving ancestry	No. of RV sets in AM model (correlated ones removed)	Proportion range of local ancestry variance explained by burden scores (R ^2^ )	Proportion range of local ancestry variance explained by the top-associated burden score (R ^2^ )
N2-acetyllysine		Negative Control	2	Afr	7	(0.04, 0.19)	(0.003, 0.08)
N-acetylarginine		Test Regions	2	Afr	1	(−0.04, 0.03)	—
3-aminoisobutyrate		Test Regions	5	Amer	1	(−2.71E-04, 0.01)	—
5-oxoproline		Test Regions	8	Afr	1	(−0.04, 0.01)	—
Arachidonoylcholine		Test Regions	11	Amer	1	(−0.07, 0.004)	—
1-methylimidazoleacetate		Test Regions	12	Amer	1	(−0.01, 0.02)	—
Ethylmalonate		Test Regions	12	Afr	1	(−0.17 0.14)	—
Propyl 4-hydroxybenzoate sulfate		Test Regions	16	Afr	13	(−0.01, 0.31)	(−0.06, 0.09)
Cys-gly, oxidized		Test Regions	16	Amer	1	(−0.02, 0.04)	—

We note that the small sample size in batch 1 (n = 3842) could reduce power to detect RV associations compared to biobank-scale studies. Our analysis may therefore have missed RV sets with lower significance levels that are indeed associated with the metabolite. There could be an RV set (or multiple sets) that we did not detect due to low power, but such a low-powered set is unlikely to explain a substantial proportion of the AM signal. However, we acknowledge that there could be a scenario in which many rare variant sets each explains a low proportion of variance but together they explain a lot. Admixture mapping can tag variants with ancestry-specific effects that may be missed by GWAS, so examining ancestry-enriched but GWAS non-significant variants is also important—but requires replication. Note that we only identified significantly associated genetic variants, rather than studying multiple causal variants in this framework, which requires fine-mapping techniques and could be included in future work.

This work aimed to explain the previously unexplained AM signals in association with metabolites levels by 1) including the sets of RVs through an adapted detection pipeline, and 2) expanding the genomic regions in search for common variants to account for the long LD at the local ancestry levels. After adjusting for common variants, the significant AM signals in all twelve previously unexplained genomic regions were largely attenuated. While RV sets explained only a small portion of the AM signals, they were often located within the same genes or in close genomic proximity to the associated common variants, highlighting potential interactions between rare and common genetic variation. Importantly, this study represents one of the first efforts to systematically evaluate RVs within the framework of admixture mapping, providing new insight into the genetic architecture underlying AM signals and offering a generalizable framework for future studies in admixed populations.

## Materials and methods

### The HCHS/SOL

The Hispanic Community Health Study/Study of Latinos (HCHS/SOL) is a multi-center, population-based prospective cohort study that enrolled individuals of self-identified Hispanic/Latino background aged 18–74 years between 2008 and 2011 [33]. The primary objective of the study is to investigate the prevalence and determinants of chronic conditions, including cardiovascular disease, asthma, and sleep disorders within Hispanic/Latino populations [33]. Participants were recruited from four U.S. field centers located in the Bronx (New York), Chicago (Illinois), San Diego (California), and Miami (Florida) [12, 33, 34]. Each study participant self-reported their Hispanic/Latino background, with major groups including Mexican, Puerto Rican, Cuban, Dominican, Central American, and South American [34, 35]. Baseline data collection included demographic information (sex and age at the time of participant’s clinic visit), clinical measurements such as body mass index (BMI), and estimated glomerular filtration rate (eGFR) [33]. The eGFR was calculated from serum cystatin C concentrations using the Chronic Kidney Disease Epidemiology Collaboration equation, excluding demographic variables [36].

### Local ancestry inference

FLARE v0.3.0 was used for local ancestry inference (LAI), as previously reported. HCHS/SOL individuals were genotyped using the Multi-Ethnic Genotyping Array (MEGA) [37], as part of their involvement in the Population Architecture Using Genomics and Epidemiology (PAGE) consortium [38]. Genotypes were subsequently imputed to the TOPMed v1.0 reference panel [39]. Both the reference haplotypes and HCHS/SOL variant data were phased with Beagle v5.4 [40]. SNPs were kept if they had a minor allele frequency ≥ 0.005 and, for imputed variants, an imputation quality score (R^2^) > 0.8, providing a stringent yet efficient quality threshold [13, 41]. All FLARE-derived coordinates are reported in GRCh38 (hg38). The reference haplotypes were generated from the Human Genome Diversity Project (HGDP) data [42, 43], which were derived from whole-genome sequencing data of 220 participants across three geographic regions: Central-South America (61), Europe (155), and Sub-Saharan Africa (104). The FLARE-inferred local ancestry dataset comprises 11 928 HCHS/SOL individuals and 3 618 751 SNPs [37]. Note that admixture mapping in this paper was performed on individuals with local ancestry information from the FLARE dataset who also had WGS data and were included in both metabolomic batches (n = 5307).

### Selected AM association regions

The previously published AM study identified significant associations between metabolites and local Amerindian or African ancestry in the HCHS/SOL cohort [19]. To serve as negative controls, we selected four genomic regions where significant AM signals were found in association with metabolites (N2-acetyllysine, betaine, N-acetylglucosaminylasparagine, and cysteinylglycine) and could be attributed to common variants. In addition, we selected twelve test regions where the significant signals with the metabolites (N-acetylarginine, 3-aminoisobutyrate, 5-oxoproline, N-acetylputrescine, carnitine, 3beta-hydroxy-5-cholestenoate, arachidonoyl choline, 1-methylimidazoleacetate, ethylmalonate, N-acetylcarnosine, propyl 4-hydroxybenzoate sulfate, and cys-gly, oxidized) were not explained by common variants.

### The modified STAAR pipeline

We adapted the STAAR pipeline [44, 45] to identify RV sets that might explain these unexplained AM signals, using specific genomic intervals rather than genome-wide testing (Fig. 1). The metabolite-associated AM regions were identified in the previous work on the level of genomic intervals [19]. We defined genomic association regions with AM signal as all consecutive local ancestry intervals with genome-wide significant AM associations with the metabolite of interest (including multiple adjacent intervals in addition to the most significant interval) (Table 1). For AM signals that only span one local ancestry interval, we expanded the interval by a ± 1 megabase window for analysis, or until reaching the start or end of the chromosome, whichever occurred first. Sets of RVs (MAF < 0.01) were then constructed through the adapted pipeline [4]. We performed gene-centric RV set associations for coding and non-coding functional categories (Fig. 1). STAAR employs burden tests, variance component tests, with a multi-dimensional omnibus weighting framework to incorporate various variant-annotation scores and qualitative functional categories for genetic variants. While the result of different tests were reported, we only focused on the STAAR burden (STAAR-B) and the STAAR omnibus (STAAR-O) test result to evaluate whether or not a RV set has significant association with the metabolite. We modified the STAAR pipeline to extract burden scores, which could be combined with the local ancestry association in a conditional model. Under the framework of using conditional analysis with both local ancestries and RVs in the same model, we need to extract an effect size of the local ancestry dosage while adjusting for the RV set. This is impossible when using a variance component test such as SKAT, as it does not allow for estimation of the impact of the variant set on the AM-metabolite association. Therefore, we used the weights from the burden tests in STAAR to generate Burden scores, which were then used as covariates in the conditional admixture mapping analysis.

### Common variant associations

For each metabolite, we first fit a linear mixed ‘null’ model, i.e. without any of the variants, adjusting for age, sex, the first 5 genetic PCs, self-reported backgrounds, eGFR, and incorporating random effects for household, census block, and genetic relatedness (kinship matrix).

In each defined genomic region, we first performed single-variant association tests in batch 1 to identify significantly associated common variants (*P* < 5 × 10^−8^). Among these genome-wide significant common variants, we then performed conditional analyses adjusting for common variants previously associated with each metabolite in HCHS/SOL, as reported in the GWAS Catalog [20], to identify additional associated common variants (P_cond_ < 0.001) that are independent of the reported GWAS hits. For all common variants passing the P_cond_ threshold, LD-pruning was performed using the LD_pruning() function from the STAARpipelineSummary package (cond_p_thresh = 1 × 10^−4^, method_cond = ‘optimal’) [44]. After pruning the newly associated variants (independently of the catalog variants), they were combined with the catalog-reported common variants, with no additional pruning of the merged set, to generate the final ‘associated common variants’ set for each metabolite. This set was then used in subsequent conditional analyses of RVs and in conditional AM (Fig. 1). We also calculated the span (maximum distance in base pairs) of the ‘associated common variants’ within each genomic region.

To assess whether the pruning order affected the conditional admixture mapping results, we generated a second set of associated common variants in which the pruning step is performed on the combined list of reported variants and those passing the P_cond_ threshold into the final set (STAAR cond_p_thresh = 1 × 10^−4^ as in the main analysis, Supplementary Data).

### Rare variants testing in the modified STAAR pipeline

Rare variant association analysis was conducted separately for the two batches, using all individuals with available WGS and local ancestry data (3842 in batch 1). STAAR pipeline uses multiple burden tests for each set of RVs, with weights based on ranks from functional annotations, as well as on variant frequencies via the beta distributions with different parameters, to upweight RVs [45]. Protein-coding genes were selected by Ensembl and non-coding RNA (ncRNA) genes selected by GENCODE [44]. The coding functional categories include plof (putative loss of function), plof_ds (putative loss of function or disruptive missense), missense, disruptive missense, synonymous, ptv (protein-truncating variant), and ptv_ds (protein-truncating variants or disruptive missense). The non-coding categories include downstream, upstream, UTR (untranslated region), CAGE (cap analysis of gene expression)-defined promoters and enhancers, DHS (DNase I hypersensitive site) defined promoters and enhancers.

Using the modified pipeline, we extracted all burden scores from the default STAAR burden tests and evaluated their pairwise correlations, retaining the smallest set of burden scores that were not highly correlated (r < 0.85). Because the burden scores generated under the two Beta weightings were highly correlated, only one of the Beta (1,1) or Beta (1,25) burden scores served as the final covariate in the conditional AM model (Fig. 1). Because rare variant set composition differs between batches, we chose a correlation threshold of 0.85 for burden scores in the discovery stage to retain more rare variant sets for replication testing. Some RV sets are nested, for example, plof_ds comprises both plof and disruptive missense variants, so we deduplicated RV set selections in the AM model.

We used Bonferroni correction to define statistically significant burden scores in unconditional RV association analysis calculated separately for coding (*P* < 2.52 × 10^−5^ for batch 1 and *P* < 2.71 × 10^−5^ for batch 2) and non-coding (*P* < 1.63 × 10^−5^ for batch 1 and *P* < 1.47 × 10^−5^ for batch 2) analyses based on the number of tests performed in each gene and functional category. A variant set is considered statistically significant in association with the metabolite only if its *P*-value is below the computed thresholds for any of the STAAR-O or STAAR-B (1, 25) omnibus *P*-values. For regions in which significant RV sets were detected, we also performed conditional analysis where the associated common variants were adjusted for in new models testing RV associations. In these conditional analyses that association *P*-value threshold was 0.001, such that RV sets with conditional *P*-value < 0.001 were carried forward for conditional AM analysis. We used this liberal threshold to allow for more RV sets to potentially explain the AM signals.

### Conditional admixture mapping with identified variants

After identifying associated common variants and obtaining the burden scores from the RV sets, we fitted the AM model in four ways: first without any genetic variants, only with the ‘associated common variants’, only with the RV sets, and finally with both the associated common variants and the RV sets. This approach allowed us to see whether the local ancestry associations became statistically insignificant, thus revealing if these variants accounted for the observed associations. The AM conditional analyses are then conducted in the replication (batch 2, N = 1465) dataset for validation. The significance threshold in AM using FLARE local ancestries was previously estimated using STEAM [37], which quantifies the hypothesis testing burden in AM based on ancestry correlation structure and the number of generations since admixture [46]. For the AM sensitivity analysis, we repeated the conditional AM using the jointly pruned common variants (listed in Supplementary Data).

### Variance explained between local ancestry and adjusted common or RVs

To characterize the correlation between local ancestry and the genotypes of the common variants and RV burden scores included as covariates in the conditional admixture mapping models, we regressed local ancestry dosage on each of these terms. Specifically, for each of the 16 regions with a significant null-model metabolite–local ancestry association (*P* < 2.58 × 10^−6^), we randomly sampled 50 SNP-level local ancestry counts (seed = 1) to serve as outcomes. Each model included the common variant genotypes or rare-variant burden scores as the exposure, all covariates used in the corresponding conditional admixture mapping model (Fig. 1), and random effects for household, census block, and kinship. We repeated this procedure using the lead common variant or the lead rare-variant burden score, defined as the variant or RV set most significantly associated with the metabolite in the unconditional single-variant analysis.

For each setting, we quantified the proportion of local ancestry variance explained by the exposure as the relative reduction in total residual variance between the reduced model (covariates and random effects only) and the full model (reduced model plus the exposure term):


$$\mathrm{R}^2=1-\frac{\sum_k{\hat{\sigma}}_{k,\mathrm{full}}^2}{\sum_k{\hat{\sigma}}_{k,\mathrm{reduced}}^2}$$

where ${\hat{\sigma}}_k^2$denotes the *k*-th estimated variance component (household, census block, kinship, and residual error) from the respective fitted model. This quantity approximates R^2^, reflecting the fraction of local ancestry variation captured by the exposure after accounting for all other model terms.

## Supplementary Material

Supplementary_material_ddag098

## Data Availability

The HCHS/SOL fully supports data sharing for HCHS/SOL–approved manuscript proposals with outside investigators. All data sharing is conducted in accordance with HCHS/SOL study and NIH policies and governed by a Data and Materials Distribution Agreement (DMDA) between UNC and the external institution, ensuring the confidentiality and privacy of HCHS/SOL participants and their families. Alternatively, de-identified HCHS/SOL data are publicly available at BioLINCC and dbGaP for the subset of the study cohort who authorized general use of their data at the time of informed consent. HCHS/SOL WGS data are available by application to dbGaP according to the study-specific accession ‘phs001395’. Phenotype data are also available from dbGaP according to the study-specific accession ‘phs000810’.

## Code availability statement

The code for adapted STAAR pipeline, conditional AM, and figures and tables are available at the github repository: https://github.com/xc448/HCHS_SOL_Rare_Variants
